# Immediately repaired penile fractures: age is the only predictor of postoperative long-term functional outcomes

**DOI:** 10.1093/sexmed/qfad048

**Published:** 2023-08-29

**Authors:** Ibrahim Erkut Avci, Hasan Yilmaz, Naci Burak Cinar, Enes Malik Akdas, Kerem Teke, Mustafa Melih Culha

**Affiliations:** Department of Urology, School of Medicine, Kocaeli University, 41380, Kocaeli, Turkey; Department of Urology, School of Medicine, Kocaeli University, 41380, Kocaeli, Turkey; Department of Urology, School of Medicine, Kocaeli University, 41380, Kocaeli, Turkey; Department of Urology, School of Medicine, Kocaeli University, 41380, Kocaeli, Turkey; Department of Urology, School of Medicine, Kocaeli University, 41380, Kocaeli, Turkey; Department of Urology, School of Medicine, Kocaeli University, 41380, Kocaeli, Turkey

**Keywords:** Penile fracture, Immediate repair, Erectile dysfunction, Postoperative complications, Functional outcomes

## Abstract

**Background:**

Penile fractures can lead to many functional complications, especially erectile dysfunction (ED). Few studies have evaluated the factors that predict late complications of an immediately repaired penile fracture.

**Aim:**

To identify the potential predictors of long-term poor functional outcomes following immediate surgical intervention for penile fractures.

**Methods:**

Sixty-eight consecutive patients with suspected penile fracture between 2003 and 2022 were retrospectively reviewed. Functional outcomes, postoperative complications, and follow-up duration were obtained from the records of follow-up visits. Age at presentation, location and length of the tunical tear, the presence of urethral rupture, and time to surgery were all analyzed as potential risk factors for postoperative functional outcomes.

**Outcomes:**

Postoperative erectile function and intercourse satisfaction were measured by the IIEF-5 (the 5-item version of the International Index of Erectile Function). Penile curvature, a palpable nodule, and paresthesia/numbness were detected by physical examination. Uroflowmetry was used to assess urinary flow in patients who underwent urethral repair.

**Results:**

Fifty-eight patients were analyzed. The mean ± SD age was 38.1 ± 10.4 years; the median follow-up was 79.0 months (range, 13-180); the median time to surgery was 9.8 hours (4-30); and the median tunical tear length was 15.5 mm (4-40). Urethral rupture was observed in 8 patients (13.8%). In univariable analyses, urethral rupture was associated with postoperative complications (*P* = .034). In addition, age at presentation and tunical tear size were significantly associated with postoperative complications and ED (*P* < .05). However, in multivariable analyses, only age at presentation significantly predicted postoperative complications and ED (*P* = .004 and *P* = .037).

**Clinical Implications:**

Age at presentation is the most important factor determining the prognosis of immediate surgical repair of the penile fracture, which aids in predicting potential complications and discussing them with patients prior to surgical intervention and during the follow-up period.

**Strengths and Limitations:**

The study’s retrospective design is an important limitation. Furthermore, there were no data on an IIEF-5 outcome measuring preoperative erectile function.

**Conclusion:**

These results revealed an association between (1) urethral rupture, longer tunical tears, and older age and (2) the development of late complications. The remarkable finding of this study was that age at presentation was the only significant predictor of functional complications based on multivariable analyses. This relationship also remained robust in tests evaluating the covariance of the effects of aging on ED.

## Introduction

Penile fracture is defined as the formation of a tear in the tunical albuginea as a result of penile trauma that occurs while the penis is erect.^1^ The management of penile fractures is based on data obtained from retrospective case series due to their rarity. Tunical tears can either heal spontaneously or be surgically repaired.^2^ However, complications were more common when a conservative approach was chosen.^3^ Thus, immediate surgical repair is currently recommended.^4-7^ Erectile dysfunction (ED), penile curvature, penile plaque, and painful erection are the most common complications following penile fracture.^4^^,^^5^^,^^8^ Numerous studies have reported long-term functional outcomes or complication rates following immediate surgical repair, yet only a few studies have been conducted to identify predictors of these outcomes.^9-13^

In previous studies, older age (>50 years), delayed presentation, proximal tunical tear location, bilateral involvement, and a larger size of the tunical tear were associated with ED.^9-13^ Indeed, the conclusions of these studies were primarily based on a simple univariate analysis in which each independent variable is tested separately. Identifying the adjusted relationship between the outcome variable and multiple potential predictive factors is essential.^14^ There is a lack of studies in the literature to evaluate the adjusted effects of possible predictive factors on the late functional complications of penile fracture repairment. We hypothesized that age at the fracture time, tunical tear location and laterality, fracture etiology, patient educational level, delayed operation time, and concomitant urethral rupture would be associated with late complications and poor functional outcomes of penile fractures. We aimed to examine the potential interaction among these factors and determine the independent predictive factors on the complications and poor functional outcomes after immediate surgical repair of penile fractures.

## Methods

### Patients

**Figure 1:**
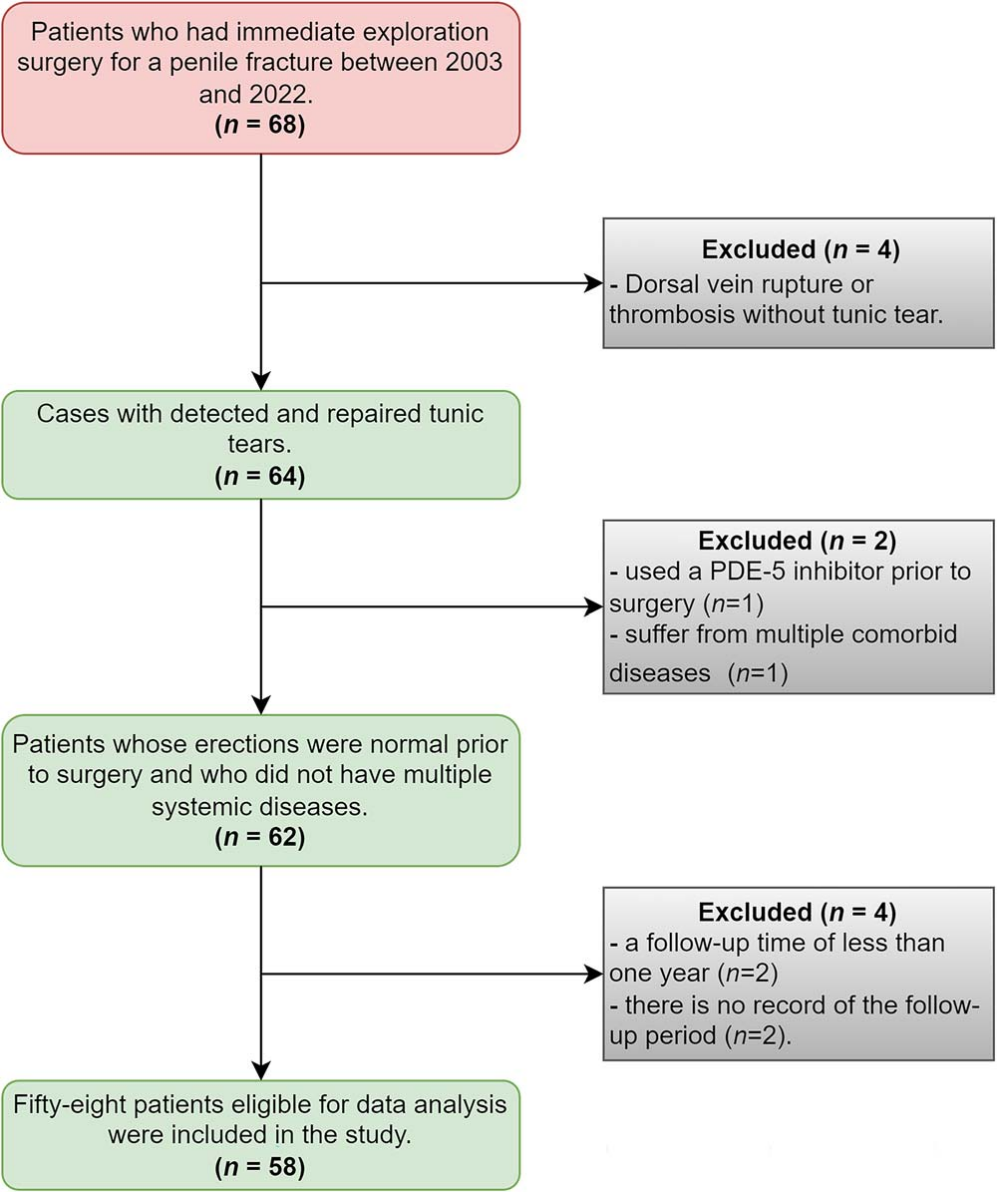
Study design.

Medical records were retrospectively reviewed for 68 consecutive patients who underwent immediate penile exploration for a suspected penile fracture at a single tertiary referral center (Kocaeli University) between January 2003 and February 2022. The protocol of this retrospective cohort study was approved by the Kocaeli University Clinical Research Ethics Committee (GOKAEK-2022/20.07), and informed and signed consent was obtained from all patients prior to surgery. Data sources consisted of medical records from the emergency and urology departments and the urology outpatient clinic covering the follow-up period. Exclusion criteria were as follows: being treated for ED prior to the operation; having no tear in the tunica albuginea during penile exploration; and experiencing multiple comorbid diseases or conditions that are common risk factors for ED, such as diabetes mellitus, dyslipidemia, hypertension, cardiovascular disease, obesity, metabolic syndrome, the use of psychotropic drugs, chronic kidney disease, rheumatic disease, and stroke. Furthermore, cases with no follow-up visits or follow-up <1 year were excluded from the study (Figure 1).

The following information was retrieved from emergency room patient charts: age at presentation of the penile fracture, education level, trauma etiology, symptoms and clinical findings, erectile and sexual function, medications, and comorbid diseases, as well as the evaluation of a consulted urologist. Additional data were collected as follows: time of the surgical exploration; physical status classification system score (American Society of Anesthesiologists); presence, length, and location of tunical tears; presence of urethral ruptures; length of hospitalization; and time between discharge and resumption of sexual activity.

The follow-up visits included multiple assessments: medical and sexual history; physical examination, including evaluation of penile curvature, palpable nodule, and paresthesia/numbness; specific diagnostic tests for ED, if needed; and for patients undergoing urethra repair, information concerning lower urinary tract symptoms and evaluation of urinary flow with uroflowmetry. If a Qmax <15 mL/s was detected, it was considered suspicious of stenosis, and urethroscopy was performed. All patients completed the Turkish-validated 5-item version of the International Index of Erectile Function (IIEF-5) at each visit during the follow-up period.^15^^,^^16^ ED was defined as follows: an IIEF-5 score <12 or a score classified as mild or mild-moderate ED with clinically confirmed ED (ie, specific diagnostic tests for ED or receipt of ED-specific treatment). An IIEF-5 score >21 at the last visit or a score classified as mild ED without clinically confirmed ED (ie, no ED treatment or diagnosis) was considered normal erectile function. Functional outcome assessment focused on development of ED, sexual intercourse problems, or urethral stricture.

### Surgical technique

In all patients, a subcoronal incision with circumcision was used to expose the tunica albuginea. After evacuation of the hematoma to reveal the tunical tear, the lips of the defect were brought together via forceps, and the length of the incision was determined by the distance between the ends of the defect, as measured with a disposable surgical ruler. The defect was then repaired with interrupted absorbable polyglactin sutures. An artificial erection with saline was performed after repair of the tunical defect to rule out the presence of other defects. Before the tunical defect was repaired, the spongious body of the urethra was always inspected for possible traumatic injury concomitant to penile trauma. Cystoscopy was performed in case of suspected urethral injury. If a urethral defect was identified, a simultaneous repair was performed. A compressive dressing was always left in place and removed on the first postoperative day. After discharge, patients were given oral antibiotics for 1 week and oral nonsteroidal anti-inflammatory drugs for 2 weeks; those who had urethral repair continued oral antibiotic therapy for as long as they needed (~2-3 weeks) to remain catheterized with urethral or suprapubic catheterization. They were also advised not to engage in sexual activity for 6 weeks after discharge.

### Statistical analyses

All data were analyzed with SPSS version 21 (IBM). Descriptive statistics were used in the analyses of quantitative data. The Kolmogorov-Smirnov test was conducted to test the normal distribution of continuous variables. The Student *t*-test was performed to compare parametric data, while the Mann-Whitney *U* test was used for nonparametric data. The chi-square test was utilized to compare categorical variables.

Patients were divided into 2 groups: any postoperative complication (ED, urethral stricture, penile curvature, palpable nodule, or paresthesia/numbness) and no complications. Post hoc power analyses were performed to compute the retrospective power of the observed effect based on the sample size and the differences between two means or proportions parameters of our data set. G*power software (version 3.1.9.7) was used for power analysis.

Multivariable logistic regression analysis tested potential risk factors that could predict the likelihood of postoperative complications. Furthermore, these factors were analyzed as possible predictors of the development of ED. Hierarchical multiple regression analysis and analysis of covariance (ANCOVA) were conducted to test the covariance role of the aging on the development of ED. In the hierarchical multiple regression analyses, 2-step block analyses were performed to control for the effect of age at the time of ED diagnosis on the relationship between patient age at the time of fracture presentation and IIEF-5 score. The first block included age at diagnosis of ED as a predictor and IIEF-5 score as a dependent variable. In the second block, age at presentation was added as a predictor. In the ANCOVA, covariance analyses were performed to control for the effect of age at diagnosis of ED on the relationship between age at presentation and postoperative ED. Finally, age at presentation and reference binary outcome variables of the presence of ED or postoperative complications were used to plot a receiver operating characteristic (ROC) curve. ROC curve analyses were performed with MedCalc (trial version 20.215). The area under the curve summarized discriminative ability with pairwise testing by the DeLong test. The value with the highest sensitivity and specificity was selected for the cutoff. *P* < .05 was considered significant in all statistics.

## Results

Fifty-eight patients were included in the analyses (Figure 1). Patient demographics and clinical and intraoperative findings are summarized in Table 1. The mean ± SD age was 38.09 ± 10.44 years (range, 18-61). All patients reported normal erectile function before the fracture, and almost none had comorbidities. In the majority (70.7%), the etiologic cause of penile fracture was sexual intercourse. Immediate surgical repair was performed for all, and the median time between fracture and surgical repair was 9.8 hours (IQR, 5.5-12.6). The median length of tunical tears was 15.5 mm. Tunical tear was localized significantly more often on the right side (*P* = 0.013), ventrally (*P* < .001), and proximally (*P* < .001) than in other locations. Urethral rupture occurred in 8 patients (13.8%), and 4 (6.9%) had complete urethral disconnection with bilateral tunical tears.

**Table 1:** Patient demographics and clinical and intraoperative findings.

	**No. (%)** ^**a**^
Age, years, mean ± SD	38.09 ± 10.442
Level of education	
Primary/secondary	28 (48.3)
High school/university	30 (51.7)
ASA physical status classification system	
1	53 (91.4)
2	5 (8.6)
Etiology	
Sexual intercourse	41 (70.7)
Forceful bending of the erect penis	7 (12.1)
Rolling over in the bed	6 (10.3)
Blunt trauma	4 (6.9)
Symptoms and clinical findings	
Crackling sound	43 (74.1)
Detumescence	43 (74.1)
Pain	47 (81.0)
Hematoma	53 (91.4)
Swelling and edema	55 (94.8)
Urethral bleeding	5 (8.6)
Time until the operation, h, median (IQR)	9.8 (5.5-12.6)
Location of the tunical tear	
Right	36 (62.1)
Left	17 (29.3)
Bilateral	5 (8.6)
Ventral	53 (91.4)
Dorsal	5 (8.6)
Proximal	38 (65.5)
Midshaft	11 (19.0)
Distal	9 (15.5)
Tunical tear size, mm, median (range)	15.5 (4.0-40.0)
Urethral rupture	
Partial	4 (6.9)
Complete	4 (6.9)
Hospitalization time, days, median (IQR)	1 (1-2)

Abbreviation: ASA, American Society of Anesthesiologists.

a Data are presented as No. (%) unless noted otherwise.

Follow-up time and functional results are given in Table 2. The median follow-up time was 79.0 (range, 13-180 months). The median time to sexual intercourse after the repair was 6 weeks (IQR, 5-7). According to the IIEF-5, 75.9% (n = 44) of patients had normal erectile function, and those with mild ED (n = 5) did not report any ED-related discomfort and did not require phosphodiesterase type 5 inhibitor treatment during the follow-up period. These 49 patients were defined as having no ED. Patients with mild to moderate ED (IIEF-5 score, 12-16) underwent specific diagnostic tests for ED or received ED-specific treatment. Thirty-one patients did not suffer any postoperative complication; however, at least one functional complication was observed in the remaining 27. A palpable nodule was most commonly found (13.8%) in physical examination. Penile curvature was detected in 7 patients (12.1%). These did not exceed 30° and did not require surgery. Among patients with urethral rupture, one patient underwent cystoscopy because of a Qmax <15 mL/s, but this was associated with benign prostatic obstruction.

**Table 2:** Long-term functional and complication outcomes.

	**No. (%)** ^**a**^
Follow-up, mo, mean ± SD	78.97 ± 41.019
Elapsed time to sexual activity, wk, median (IQR)	6 (5–7)
IIEF-5 score after the operation, median (IQR)	22 (21–23)
Erectile dysfunction severity: IIEF-5 classification	
22-25: no ED	44 (75.9)
17-21: mild	5 (8.6)
12-16: mild to moderate	6 (10.3)
8-11: moderate	1 (1.7)
5-7: severe	2 (3.4)
Postoperative complications	
None	31 (53.4)
Erectile dysfunction	9 (15.4)
Penile curvature	7 (12.1)
Palpable nodule	8 (13.8)
Painful erection	6 (10.3)
Paresthesia/numbness	3 (5.2)

Abbreviations: ED, erectile dysfunction; IIEF-5, 5-item International Index of Erectile Function.

a Data are presented as No. (%) unless noted otherwise.

Comparative outcomes of patients according to postoperative complications are presented in Table 3. Age at presentation was greater in the complication group (42.26 ± 10.387 vs 34.45 ± 9.183 years, *P* = .004). The age parameter’s power (1 – β error probability) was 0.9057 (effect size, *d* = 0.7966; critical *t* = 1.702) with an error of 0.05. No statistical difference was found in terms of education, American Society of Anesthesiologists grade, delaying time before operation, hospitalization time, elapsed time to sexual activity, or follow-up time (*P* > .05). The IIEF-5 score was significantly higher in the no-complication group (*P* = .002). Tunical tear size was significantly higher (*P* = .004) and bilateral (*P* = .018) in the complication group. The power for the tunical tear size parameter was 0.8708 (effect size, *d* = 0.8278; critical *t* = 2.003) with an error of .05. The majority of urethral ruptures were observed in the complication group (7/8, *P* = .020). The power for the presence of urethral ruptures was 0.7103 (effect size, critical *z* = 1.959) with an error of .05.

**Table 3:** Comparative outcomes of those with and without ≥1 complications during follow-up^a^

	**Without postoperative complications (n = 31)**		**With postoperative complications (n = 27)**		
**Variable**	**No. or median**	**% or IQR**	**No. or median**	**% or IQR**	***P* value**
Age, y, mean ± SD	34.45	9.183	42.26	10.387	**.004** ^b^
Low education level	17	54.8	11	40.7	.284 ^c^
ASA 1	28	90.3	25	92.6	.758 ^d^
Sexual etiology	24	77.4		17 63.0	.288 ^c^
Time until the operation, h	9.5	7-12	10	5.5-13.5	.956 ^e^
Tunical tear					
Proximal	21	67.7	17	63.0	.702 ^c^
Dorsal	4	12.9	1	3.7	.359 ^d^
Presence of					
Bilateral tunical tear	0	0	5	18.5	**.018** ^d^
Urethral rupture	1	3.2	7	25.9	**.020** ^d^
Tunical tear size, mm	11	9.0-20.0	20	15.0-25.0	**.004** ^e^
Hospitalization time, d	1	1.0-2.0	2	1.0-2.0	.788 ^e^
Elapsed time to sexual activity, wk	6	5.0-8.0	6	4.0-7.0	.431 ^e^
Follow-up, mo, mean ± SD	87.84	40.263	68.78	40.206	.077 ^b^
IIEF-5 score	23	22.0-24.0	22	16.0-22.5	**.002** ^e^

Abbreviations: ASA, American Society of Anesthesiologists; IIEF-5, 5-item International Index of Erectile Function.

a Data are presented as No. (%) or median (IQR) unless noted otherwise. b Independent Sample T Test, c Pearson Chi-Squared, d Fisher’s Exact Test, e Mann-Whitney U. The bold *p* values indicate a *p* < 0.05, that is a statistically significant difference.

The univariable and multivariable logistic regression analyses of the association between possible predictive factors and the probability of ED or at least 1 postoperative complication are given in Table 4. In univariable analyses, age at presentation of the penile fracture and tunical tear size were significant predictors for ED and postoperative complications (*P* < .05). Additionally, urethral rupture was associated with postoperative complications (*P* = .034) in univariable analyses. However, in multivariable analyses, only age at presentation was an independent risk factor for ED and postoperative complications (odds ratio, 1.18 [95% CI, 1.05-1.31; *P* = .004]; odds ratio, 1.07 [95% CI, 1.01-1.14; *P* = .037]).

**Table 4:** Univariable and multivariable logistic regression analysis: association between possible predictive factors and erectile dysfunction or any postoperative complication.

	**Erectile dysfunction**				**Postoperative complications**			
	**Univariable**		**Multivariable** ^**a**^		**Univariable**		**Multivariable** ^**b**^	
**Variable**	***P* value**	**OR (95% CI)**	***P* value**	**OR (95% CI)**	***P* value**	**OR (95% CI)**	***P* value**	**OR (95% CI)**
Age, y: continuous	**.002**	1.19 (1.07-1.32)	**.004**	1.18 (1.05-1.31)	**.007**	1.09 (1.02-1.15)	**.037**	1.07 (1.01-1.14)
Education level: low vs high	.803	0.83 (0.20-3.48)			.285	0.57 (0.20-1.61)		
Etiology sexual vs others	.613	1.54 (0.29-8.33)			.231	0.50 (0.16-1.56)		
Time until the operation, h: continuous	.583	1.03 (0.92-1.16)			.803	0.99 (0.90-1.08)		
Location of the tunical tear								
Proximal vs mid/distal	.497	0.61 (0.14-2.57)			.703	0.81 (0.27-2.40)		
Dorsal vs ventral	.773	1.41 (0.14-14.26)			.241	0.26 (0.03-2.48)		
Bilateral tunical tear: presence vs absence	.139	4.38 (0.62-31.04)			.995	1.00 (0.24-8.89)		
Urethral rupture: presence vs absence	.081	4.40 (0.83-23.29)			**.034**	2.35 (1.20-8.91)	.421	2.89 (0.22-38.34)
Tunical tear size: continuous, mm	**.045**	1.09 (1.01-1.18)	.361	1.05 (0.95-1.16)	**.006**	1.11 (1.03-1.20)	.159	1.07 (0.97-1.18)

Abbreviation: OR, odds ratio. Bold indicates *P* < .05.

a Omnibus < 0.001, Hosmer and Lemeshow = 0.964, percentage correct = 89.7%, Nagelkerke *R*^2^ = 0.423.

b Omnibus = 0.002, Hosmer and Lemeshow = 0.938, percentage correct = 70%, Nagelkerke *R*^2^ = 0.307.

A hierarchical multiple regression with a block of variables was performed to test the predictions. As a result of the model in which the age at diagnosis of ED was a control variable, age at presentation was still significantly associated with the IIEF-5 score (*b* = −1.23, *t* = −4.375, *P* < .001). Additionally, we utilized covariance analyses to control the effect of age at ED diagnosis on the relationship between age at presentation and postoperative ED. After controlling for the effect of age at ED diagnosis, age at presentation still had a significant effect on the development of postoperative ED (ANCOVA; *F*_1,55_ = 18.57, *P* < .001).

**Figure 2:**
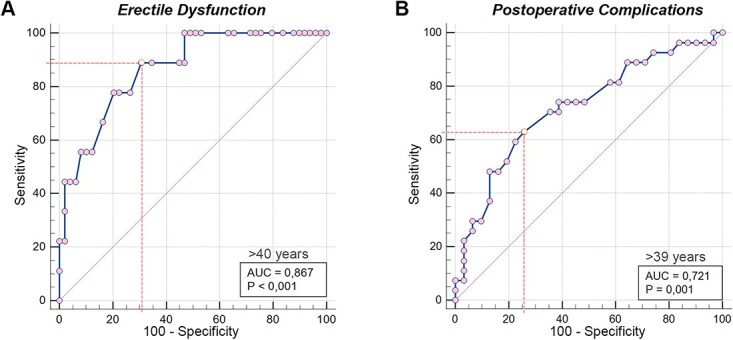
Receiver operating characteristic curves that predict postoperative erectile dysfunction and complications based on patient age at presentation of the penile fracture.

In the ROC analyses, the optimal cutoff ages were 40 years for predicting postoperative ED (88.9% sensitivity, 69.4% specificity, *P* < .001) and 39 years for predicting postoperative complications (63.0% sensitivity, 74.2% specificity, *P* = .001; Figure 2).

## Discussion

Penile fracture is a trauma, and it is usually caused by displacement of the erect penis out of the vagina during sexual intercourse, bending it between the male’s pushing force and the female’s symphysis pubis and tearing the tunica at the weakest and most exposed point.^17-19^ Sometimes penile fracture occurs with forced bending of the penis for the abrupt termination of an erection during masturbation (Taqaandan click).^20^

Penile fracture is currently considered a urologic emergency. Surgical intervention with closure of the tunica albuginea is recommended; this ensures the lowest rate of long-term complications.^21^ Comparison of immediate surgical treatment of penile fractures vs a conservative approach on postoperative complications has been investigated in several studies. Özorak et al compared patients treated conservatively (no surgery) and those who underwent operation in the first 5 hours after the trauma occurred. At a median follow-up of 6 months, no complication occurred in cases of surgery, while in the conservatively managed group, 60% of men complained of at least 1 complication.^22^ Amer et al compared immediate vs delayed surgery in their meta-analysis of penile fracture. They demonstrated significantly fewer complications with surgery performed earlier.^7^ A recent systematic review of the management of penile fractures recommended immediate repair within 24 hours of presentation.^5^ All patients in our study underwent surgery in first 24 hours, and the median time to surgery was about 10 hours.

In the current study, tunical tear occurred significantly more often on the right side (62.1%) or ventral (91.4%) and proximal (65.5%) areas. El-Assmy et al reported right-sided injury in 56.1% of patients,^9^ and Ekeke and Eke found this in 61.9%.^23^ Ateyah et al cited a tunical tear on the right proximal third of the penis in most patients (66.7%).^24^ The fact that the fracture was more common on the right side is an interesting finding, and the reason for this is unknown. We found no significant association between tunical tear localization and postoperative complications. However, a large tunical tear or urethral rupture may indicate the severity of the trauma and the extent of the cavernosal damage.^10^^,^^13^ Ortac et al stated that a large tunical tear correlated with the IIEF-5 score and may be a predictor of higher rates of ED after penile fracture surgery.^10^ In the current study, the presence of urethral rupture and the length of tunical tear were potential risk factors for postoperative functional complications in univariable analyses, but these 2 factors lost significance in multivariable analyses. This result suggested that age at the time of fracture may play an essential role in the healing of cavernosum tissue and recovery of erectile function, independent of the effect of other factors, through its powerful impact on the normal healing of repaired tissue and restoration of function.

Complications after penile fracture are not uncommon. Amer et al conducted a meta-analysis of penile fracture.^7^ They reported that 422 of 908 patients (46.4%) in the surgical group and 93 of 104 (89%) in the conservative group had complications in pooled analyses of 22 studies. Barros et al found 13% penile curvature and 14.7% ED.^8^ In a systematic review, Falcone et al noted that palpation of an indurated scar at the level of the repair was reported by one-fourth of patients and 15% complained of significant penile curvature. They also found that postoperative ED occurred in about 21% of 298 patients.^4^ Similarly, in the current study, penile curvature was present in 12.1%, penile nodule in 13.8%, and ED in 15.4%.

Four current studies in the literature evaluated the relationship between age and postoperative ED. El-Assmy et al separated their patients into groups aged ≤50 and >50 years and found that ED was significantly higher in patients aged >50 years.^9^ Similarly, Sharma et al identified age >50 years as a risk factor for postoperative ED.^12^ Ortac et al reported that patients with ED were significantly older than those without ED (52 ± 10.8 vs 35.5 ± 10.6 years) and increasing age was significantly and negatively correlated with IIEF-5 scores (*r* = −0.59, *P* = .001).^10^ Bulbul et al noted that patients with postoperative complications were older than those without complications, but this difference was not statistically significant. This could be due to their study’s small sample size. Yet, they concluded that time to penile fracture surgery and tear size were significantly associated with the development of any complications.^13^ We also found that age at presentation was greater in patients with postoperative complications vs no complications (42.26 ± 10.39 vs 34.45 ± 9.18 years). Furthermore, we evaluated possible predictive factors for postoperative functional complications using multivariable logistic regression analyses. We determined that age at presentation was the only significant predictive factor for the development of ED and postoperative complications. Aging could have influenced the development of ED during the long follow-up period of the study. Therefore, it was necessary to determine whether the predictive effect of age at presentation of penile fracture on the development of ED was independent from aging. To demonstrate this, we utilized the ANCOVA test and hierarchical multiple regression test. We evaluated the covariant role of age at diagnosis of ED on the relationship between age at presentation and the development of ED. We also found that the significant relationship between age at presentation and ED persisted in the ANCOVA and hierarchical regression tests. In addition, in this cohort, we concluded that 40 and 39 years were the optimal cutoffs for estimating the development of ED and postoperative complications, respectively. Thus, our study provides clear evidence of the relationship between age at presentation and functional complications.

There are some limitations of the present study. The retrospective design is one of the most important. None of the patients completed a prefracture IIEF-5 form. However, a penile fracture is possible only with a penis that is sufficiently rigid to have sexual intercourse. We excluded patients who had multiple comorbid diseases or who had used phosphodiesterase type 5 inhibitors before fracture. So, it would not be unreasonable to assume that no included patients had ED before the fracture. While 58 is a decent number of patients for a disease as uncommon as penile fracture, it should be kept in mind that the sample size for the multivariable analysis method may be small.

## Conclusions

The tunical tear was significantly longer in patients with complications. However, no significant relationship was found between the location and length of the tear and the functional outcomes in multivariable analyses. Age at presentation was the only significant predictive factor for functional complications in multivariable analyses. Furthermore, this significant relationship persisted after taking into account the role of aging by covariance testing. Multicenter randomized studies are needed to generalize our findings due to the retrospective design of the study.

## Data Availability

The data underlying this article will be shared on reasonable request to the corresponding author.
